# RETRACTION: Stem Cell Niches in Glioblastoma: A Neuropathological View

**DOI:** 10.1155/bmri/9821585

**Published:** 2025-11-19

**Authors:** BioMed Research International

RETRACTION: D. Schiffer, M. Mellai, L. Annovazzi, V. Caldera, A. Piazzi, T. Denysenko, A. Melcarne, “Stem Cell Niches in Glioblastoma: A Neuropathological View,” *BioMed Research International*, 2014, 725921, https://doi.org/10.1155/2014/725921

The above article, published online on 15 April 2014 in Wiley Online Library (http://wileyonlinelibrary.com), has been retracted by agreement between the journal section editor, Letterio S. Politi, and John Wiley & Sons Ltd. The retraction has been agreed due to a duplication in Figure 3 to a previously published article. Specifically:

Figures 3e and 3f are identical to panels 2e and 2f in [1].

The authors did not provide a response to the concerns, and after assessment by the editorial board it is retracted due to concerns with the reliability of the data.

The authors did not respond to the retraction.
